# SALM4 regulates angiogenic functions in endothelial cells through VEGFR2 phosphorylation at Tyr1175

**DOI:** 10.1096/fj.201802516RR

**Published:** 2019-06-06

**Authors:** Dong Young Kim, Jeong Ae Park, Yeomyung Kim, Minyoung Noh, Songyi Park, Eunkyung Lie, Eunjoon Kim, Young-Myeong Kim, Young-Guen Kwon

**Affiliations:** * Department of Biochemistry, College of Life Science and Biotechnology, Yonsei University, Seoul, South Korea;; † Department of Biological Sciences, Korea Advanced Institute for Science and Technology, Daejeon, South Korea;; ‡ Department of Molecular and Cellular Biochemistry, School of Medicine, Kangwon National University, Chuncheon-si, South Korea

**Keywords:** VEGF signaling, endothelial leakage, vascular permeability, ischemia reperfusion

## Abstract

Angiogenesis depends on VEGF-mediated signaling. However, the regulatory mechanisms and functions of individual VEGF receptor 2 (VEGFR2) phosphorylation sites remain unclear. Here, we report that synaptic adhesion-like molecule 4 (SALM4) regulates a specific VEGFR2 phosphorylation site. SALM4 silencing in HUVECs and *Salm4* knockout (KO) in lung endothelial cells (ECs) of *Salm4^−/−^* mice suppressed phosphorylation of VEGFR2 tyrosine (Y) 1175 (Y1173 in mice) and downstream signaling upon VEGF-A stimulation. However, VEGFR2 phosphorylation at Y951 (Y949 in mice) and Y1214 (Y1212 in mice) remained unchanged. Knockdown and KO of SALM4 inhibited VEGF-A–induced angiogenic functions of ECs. SALM4 depletion reduced endothelial leakage, sprouting, and migratory activities. Furthermore, in an ischemia and reperfusion (I/R) model, brain injury was attenuated in *Salm4^−/−^* mice compared with wild-type (WT) mice. In brain lysates after I/R, VEGFR2 phosphorylation at Y949, Y1173, and Y1212 were induced in WT brains, but only Y1173 phosphorylation of VEGFR2 was reduced in *Salm4^−/−^* brains. Taken together, our results demonstrate that SALM4 specifically regulates VEGFR2 phosphorylation at Y1175 (Y1173 in mice), thereby fine-tuning VEGF signaling in ECs.—Kim, D. Y., Park, J. A., Kim, Y., Noh, M., Park, S., Lie, E., Kim, E., Kim, Y.-M., Kwon, Y.-G. SALM4 regulates angiogenic functions in endothelial cells through VEGFR2 phosphorylation at Tyr1175.

Angiogenesis is the formation of mature vasculature from a primitive vascular network. Angiogenesis occurs when a monolayer of endothelial cells (ECs) sprouts to form capillaries (1). Endothelial tip cells lead the sprouting process. These tip cells resemble axonal growth cones in morphology and function and exhibit similar lamellipodia and filopodia structures (2). Axon guidance molecules can be used to identify EC functions, because they share patterning and guidance cues. According to Adams *et al.* (3) and Pircher *et al.* (4), the axon guidance molecule families and receptors guide growing axons and blood vessels using the same signals. Among these families, leucine-rich repeats (LRRs) are thought to recruit membrane proteins (*i.e.*, receptors) and signaling molecules to synaptic junctions (5–8). In addition, several LRR-containing molecules, including adhesion molecule with IgG-like domain 2, leucine-rich α-2-glycoprotein 1, and fibronectin leucine-rich transmembrane protein 2 (FLRT2), have functions in ECs. Adhesion molecule with IgG-like domain 2 is involved in EC survival *via* protein kinase B (AKT) activation (9). Leucine-rich α-2-glycoprotein 1 is involved in endothelial TGF-β signaling (10), and FLRT2 is required for FLRT2-UNC-5 Netrin Receptor B (UNC5B) signaling in placental labyrinth formation (11). Synaptic adhesion-like molecules (SALMs) are novel axon guidance molecules containing LRRs that are involved in synapse development and functions, including synaptic transmission and plasticity (5). Five members of the SALM family have been identified. SALM1–5 have similar domain organization, with 6 LRRs, an Ig domain, and a fibronectin type III domain on the extracellular side, followed by a transmembrane domain and a cytoplasmic region that ends with a PDZ domain–binding motif (5). VEGF signaling depends on scaffolding proteins, such as synectin, that bind to PDZ domains (12). SALM4 and SALM5 do not contain PDZ-binding domains, in contrast to SALM1–3 (5). SALM4 regulates neurite branching through mechanisms that involve lipid raft–associated proteins (13). Furthermore, the hippocampal CA1 region of the *Salm4* knockout (KO) mouse has an increased number of excitatory and inhibitory synapses (14). The role of SALM4 in ECs remains unknown but must be elucidated to understand guidance by tip cells in ECs.

Vascular sprouting and permeability are highly dependent on VEGFs and their receptors (VEGFRs), which regulate EC functions, such as proliferation, migration, and viability. VEGF-A binds VEGFR1 and VEGFR2 in ECs. Although the affinity of VEGF-A is higher for VEGFR1 than for VEGFR2, VEGFR2 has higher tyrosine kinase activity (15). Therefore, VEGFR2 is regarded as the most important receptor for VEGF-A effects in ECs. The major phosphorylation sites in VEGFR2 are tyrosine (Y) 951 in the kinase-insert domain and Y1175 and Y1214 in the C-terminal domain. The VEGFR2 signaling cascade includes Y951-SRC kinase, Y1175-ERK, Y1175-PI3K–AKT-eNOS, and Y1214–p38 MAPK (16). Regulation of VEGFR2 phosphorylation is critical for angiogenesis and vascular permeability–related diseases. Nevertheless, the regulatory mechanisms of some VEGFR2 phosphorylation sites and pathways remain poorly understood.

In the present study, we determined that SALM4 is expressed in ECs and involved in angiogenic functions through VEGFR2 phosphorylation. In addition, we investigated fine-tuned potential regulators of VEGFR2 signaling in pathologic conditions using a model of acute brain ischemia and reperfusion (I/R).

## MATERIALS AND METHODS

### Isolation and culture of umbilical cord blood mononuclear cells and HUVECs

Umbilical cord blood mononuclear cells (UCB-MNCs) were isolated from human umbilical cord blood as previously described (9). Briefly, blood samples were collected from placentae with attached umbilical cords by gravity centrifugation. This procedure was approved by the Ethics Committee at Yonsei University.

HUVECs were isolated from human umbilical cord veins as previously described (17). Briefly, the veins were cannulated, perfused with PBS to remove blood, and incubated with 250 U/ml collagenase type 2 (LS004176; Worthington Biochemical, Lakewood, NJ, USA) in PBS for 10 min at 37°C. Collagenase type 2 solution was collected and centrifuged at 1200 rpm for 5 min. The pellet was resuspended in M199 medium (HyClone, SH3025301; GE Healthcare, Waukesha, WI, USA) containing 20% fetal bovine serum (FBS), 100 U/ml penicillin, 100 μg/ml streptomycin, 3 ng/ml basic fibroblast growth factor (FGF; GF003AF-MG; MilliporeSigma, Burlington, MA, USA), and 5 U/μl heparin. HUVECs were cultured on 2% gelatin-coated dishes at 37°C in a 5% CO_2_-humidified incubator and used at passages 3–7.

### Generation of *Salm4^−/−^* mice

All mice were maintained with a C57BL/6J genetic background. *Salm4* KO (*Salm4^−/−^*) mice were produced by E. Kim in the Department of Biologic Sciences, Korea Advanced Institute for Science and Technology, as previously described in Lie *et al*. (14). A mouse embryonic stem cell clone (15252A-A12; *Salm4*/*Lrfn3*_AA12) derived from the C57BL/6N strain was obtained from Velocigene (VG15252; Regeneron, Tarrytown, NY, USA). *Salm4^−/−^* mice were then generated by homologous recombination of exons 2 and 3 of *Salm4* and β-galactosidase + neomycin in a ZEN-Ub1 cassette. To generate chimeric mice, cultured embryonic stem cells (C57BL/6N) were microinjected into C57BL/6J-Tyroc-2J4 (albino B6) blastocysts. Male chimeric mice were bred with albino B6 females (C57BL/6J-Tyroc-2J4) to generate germline-transmitted first production mice (C57BL/6J-Tyroc-2J4þC57BL/6N strain). First production mice were backcrossed to C57BL/6J for 2 to 7 generations. Wild-type (WT) and KO mice were obtained from breeding *Salm4* heterozygous mice (*Salm4^+/−^* × *Salm4^+/−^*). Only male mice were used in the assays.

### Fertility assessment

Mouse fertility was measured as previously described in Kuchmiy *et al*. (18). WT or *Salm4^−/−^* 10-wk-old female mice were mated with WT or *Salm4^−/−^* 10-wk-old male mice for 16 wk. The time intervals for each litter and the litter size for each pair were recorded. The mean number of total litters/female within the 16-wk period was calculated. Pups were counted as soon as litters were found to minimize underestimation of litter size. Pups were weaned at 21 d.

### Immunofluorescence staining of tissues and HUVECs

Tissues were fixed in 4% paraformaldehyde solution (pH 7.4) overnight at 4°C and rinsed with PBS at room temperature. Tissues were then incubated in 15% sucrose solution overnight at 4°C and transferred to 30% sucrose solution at 4°C until the tissues sank. Fixed tissues were embedded in optimal cutting temperature (OCT) compound (FSC22 clear; Leica Biosystems, Wetzlar, Germany) for 30 min at room temperature, transferred to an embedding mold filled with OCT, and frozen on dry ice. Frozen tissues were cut to a 20 μm thickness at −20°C (CM1850; Leica Biosystems), and sections on slides were immunostained. The sections were fixed in acetone at −80°C for 30 min and air dried. OCT was removed with running water. The sections were incubated in blocking solution (X0909; Agilent Technologies, Santa Clara, CA, USA) for 1 h at room temperature and incubated overnight in anti-mouse CD31 (550274, 1:100; BD Biosciences, San Jose, CA, USA), β-galactosidase (A11132, 1:200; Thermo Fisher Scientific, Waltham, MA, USA), zona occludens-1 (ZO-1; 617300, 1:100; Thermo Fisher Scientific), claudin-5 (341600, 1:100; Thermo Fisher Scientific), occludin (SC-8145, 1:100; Santa Cruz Biotechnology, Dallas, TX, USA), intercellular adhesive molecule (ICAM) 1 (SC-8439, 1:100; Santa Cruz Biotechnology), vascular cell adhesion molecule 1 (VCAM1; SC-8304, 1:100; Santa Cruz Biotechnology), glial fibrillary acidic protein (GFAP; MAB360, 1:100; MilliporeSigma), or CD11b (550282, 1:100; BD Biosciences) antibodies at 4°C. Sections were then washed with 0.1% Triton X-100 in PBS, incubated with anti-rat Alexa Fluor 594 (A21209), anti-rabbit Alexa Fluor 488 (A21206), anti-goat Alexa Fluor 488 (A11055), anti-mouse Alexa Fluor 488 antibodies (A11001, 1:200; Thermo Fisher Scientific), or DAPI (D8417; 1 μg/ml; MilliporeSigma) overnight at 4°C and then washed with 0.1% Triton X-100 in PBS.

HUVECs were fixed in 4% paraformaldehyde (pH 7.4) for 20 min at room temperature and permeabilized with 0.1% Triton X-100 in PBS at 4°C. Cells were incubated with vascular endothelial (VE)–cadherin antibody (SC-9989, 1:400; Santa Cruz Biotechnology) for 2 h at room temperature. Cells were then rinsed with PBS and incubated with anti-mouse Alexa Fluor 488 antibody (1:800; Thermo Fisher Scientific) for 60 min at room temperature. Next, cells were labeled with fibrous actin (F-actin; R415, 1:300; Thermo Fisher Scientific) and DAPI (1 μg/ml) for 30 min at room temperature. All antibodies were dissolved in antibody diluent (S3022; Agilent Technologies). Samples were analyzed using a confocal microscope (LSM 700 Meta; Carl Zeiss, Oberkochen, Germany).

### Transfection of small interfering RNAs and plasmids into HUVECs

HUVECs were transfected with control and human *SALM4* small interfering RNAs (siRNAs; 80 nM) using Lipofectamine (18324; Thermo Fisher Scientific) for 3 h. Cells were assayed 48 h after transfection. Human *SALM4* siRNAs (siSALM4s) were designed by Dharmacon (Lafayette, Co, USA) using the following sequences: si 1, 5′-GGAUGAUUGUGCCGAGACA-3′; si 2, 5′-GCAGACAACUUCAUCGCCU-3′; and si 3, 5′-GUACUGGUCUUCAUCUUCG-3′. The control siRNA (siCon) sequence was 5′-UGGUUUACAUGUCGACUAA-3′. HUVECs were transfected with pEGFP-N1 (GenBank accession U55762) and pFLAG-CMV2 (E7398; MilliporeSigma) vectors using Lipofectamine LTX and Plus reagent (15338; Thermo Fisher Scientific) for 2 h. Cells were harvested 24 h after transfection. Full-length human *SALM4*, neuropilin (*NRP*) 1, and *VEGFR2* were produced from HUVEC cDNA and subcloned into the vector. *SALM4* was cloned using *HindIII* (5′-CCCAAGCTTATGGCCATCCTCCCGTTGCTC-3′) and *KpnI* (5′-CGGGGTACCCCGGGTCCCACAGGTTCGTGGCC-3′). *NRP1* was cloned using *EcoRI* (5′-CCGGAATTCATGGAGAGGGGGCTGCCGCTC-3′) and *XbaI* (5′-TGCTCTAGATCATGCCTCCGAATAAGTACT-3′). *VEGFR2* was cloned using *ClaI* (5′-CCCATCGATATGCAGAGCAAGGTGCTGCTG-3′) and *XbaI* (5′-TGCTCTAGATTAAACAGGAGGAGAGCTCAG-3′).

### 3-(4,5-Dimethyl-2-thiazolyl)-2,5-diphenyl-2H-tetrazolium bromide assay

HUVECs were seeded at a density of 3 × 10^4^ cells/ml in 2% gelatin-coated 24-well plates and incubated overnight. Cells were then starved for 12 h in serum-free medium and treated with 40 ng/ml human VEGF-A (K0921148; Koma Biotech, Seoul, Republic of Korea). After 12 h, 3-(4, 5-dimethyl-2-thiazolyl)-2, 5-diphenyl-2H-tetrazolium bromide (MTT) (0.1 mg/ml) was added, and cells were incubated at 37°C for 3 h. Residual MTT was removed, and the crystals were dissolved by incubating with dimethyl sulfoxide:ethanol (1:1). The absorbance was measured using a spectrophotometer at 560 nm.

### EC migration assay

A wound-healing assay was performed by scratching confluent HUVECs on 35-mm dishes with micropipette tips. Medium containing 1% FBS and 40 ng/ml human VEGF-A were used, with images captured at 0 and 8 h after wounding. For quantitative analysis, 5 fields/plate were photographed, and the areas between the front lines were measured using ImageJ software (National Institutes of Health, Bethesda, MD, USA). Each assay was repeated 3 times.

The chemotactic motility of HUVECs was assayed using Transwell chambers (3422; BD Biosciences) with polycarbonate filters (8-μm pore size, 6.5-mm diameter). In brief, the lower surface of the filters were coated with 0.1% gelatin, and M199 medium containing 1% FBS and 40 ng/ml VEGF-A were added to the lower wells. The HUVECs were trypsinized and resuspended in M199 containing 1% FBS to a final concentration of 1 × 10^6^ cells/ml. A 100-μl aliquot of cell suspension was added to each of the upper wells and incubated at 37°C for 2 h. Cells were then fixed and stained with hematoxylin and eosin. Nonmigrating cells were removed from the upper surface of filters using cotton swabs. Chemotaxis was quantified by counting the cells that migrated to the lower sides of the filters using optical microscopy (×200 magnification). Ten fields were counted/assay. Each sample was assayed in triplicate, and the assays were repeated 3 times.

### *In vitro* tube formation assay

Tube formation assay was performed as previously described in Choi *et al*. (19). Briefly, 250 μl growth factor–reduced Matrigel (354230; BD Biosciences) was added to 16-mm diameter tissue culture wells and allowed to polymerize for 20 min at 37°C. HUVECs were incubated in complete M199 medium. After trypsinization, harvested cells were resuspended in 1% FBS and 40 ng/ml human VEGF-A and plated on layers of Matrigel (1.5 × 10^5^ cells/ml). Matrigel cultures were incubated at 37°C and photographed at various time points (×200 magnification). The area covered by the tube network was determined using ImageJ software.

### Isolation of lung ECs from neonatal mice

Mouse lung ECs (MLECs) were isolated from 3 postnatal (6–8 d old) WT and *Salm4^−/−^* mice for seeding in a 60-mm plate as previously described in Sobczak *et al*. (20). Mice were anesthetized by intraperitoneal injection of tribromoethanol (Avertin, 2.5%) at 125 mg/kg for cardiac perfusion with PBS. The lungs were excised, minced, and digested with 250 U/ml collagenase type 2 and 4 (LS004188; Worthington Biochemical) in PBS for 45 min. The digest was homogenized through a 14-gauge needle and then filtered through a 70-μm cell strainer (352350; BD Biosciences). The cell suspension was isolated by immunoselection with CD31-conjugated magnetic beads (11035; Thermo Fisher Scientific). Medium was changed the following day and then every other day. Fully enriched cells were further sorted using ICAM2 (553326; BD Biosciences)–conjugated magnetic beads to achieve >90% purity. VE-cadherin staining was used to confirm ECs. MLECs were cultured in endothelial basal medium 2 (CC-3156; Lonza, Basel, Switzerland) containing endothelial growth medium 2 kit (CC-4176; Lonza) and 10% FBS.

### Western blot and immunoprecipitation

HUVECs were lysed in cell lysis buffer (100 mM Tris-Cl, pH 7.4, 5 mM EDTA, 50 mM NaCl, 0.5% NP-40, 1% Triton X-100) containing a protease inhibitor cocktail (05892970001; Roche, Basel, Switzerland). Lysates were centrifuged at 14,000 rpm for 15 min, and supernatants were collected. Proteins were separated by 8% SDS-PAGE and then transferred to PVDF membranes (IPVH00010; MilliporeSigma).

For immunoprecipitation of endogenous proteins, HUVECs were lysed in 1 ml NP-40 lysis buffer (50 mM Tris-Cl, pH 8.0, 150 mM NaCl, 1% NP-40, and protease inhibitor cocktail). Cell lysates were centrifuged at 14,000 rpm for 15 min. The supernatants were immunoprecipitated with antibodies against NRP1, NRP2, VEGFR1, VEGFR2, or IgG at 4°C overnight, followed by the addition of protein A agarose beads (16-156; MilliporeSigma) at 4°C for 2 h. Immunoprecipitates were washed 3 times with lysis buffer, resuspended in SDS-PAGE sample buffer containing 2-ME, and analyzed by Western blotting.

Membranes were incubated with primary antibodies (1:1000) overnight at 4°C and with secondary antibodies (1:3000) for 45 min at room temperature. The primary antibodies, phosphorylated VEGFR2-Y951 (4991), VEGFR2-Y1175 (2478), VEGFR2 (2479), phosphorylated ERK (9106), ERK (9102), phosphorylated PI3K (4228), PI3K (4292), phosphorylated AKT (9271), AKT (9272), eNOS (9572), NRP1 (3725), and NRP2 (3366) were purchased from Cell Signaling Technology (Danvers, MA, USA). Phosphorylated VEGFR2-Y1214 (AF1766) was purchased from R&D Systems (Minneapolis, MN, USA). Phosphorylated eNOS (bs-3447R) was purchased from Bioss Antibodies (Woburn, MA, USA). VEGFR1 (ab32152) was purchased from Abcam (Cambridge, MA, USA). Green fluorescent protein (SC-9996; Santa Cruz Biotechnology), Flag M2 (F1804; MilliporeSigma), VE–protein tyrosine phosphatase (PTP) (PAB048Hu01; Cloud-Clone, Katy, Houston, USA), PTP1B (AF1366; R&D Systems), and IgG (26702; GeneTex, Irvine, CA, USA) were used for immunoprecipitation. SALM4 (PA5-20706) and β-actin (MA5-15739) were purchased from Thermo Fisher Scientific. Goat anti-rabbit horseradish peroxidase (31460; Thermo Fisher Scientific) and goat anti-mouse horseradish peroxidase (31430; Thermo Fisher Scientific) were used as the secondary antibodies. Detection was performed using ECL (32106; Thermo Fisher Scientific) according to the manufacturer's instructions.

### Matrigel plug angiogenesis assay

Six-week-old mice were subcutaneously injected with growth factor–reduced Matrigel containing 200 ng/ml mouse VEGF-A (K0921632; Koma Biotech) or FGF-2 (PMG0035; Thermo Fisher Scientific) with 10 U/ml heparin. The injected Matrigel rapidly formed a single solid gel plug. After 7 d, the skin of the mouse was pulled back to expose the Matrigel plug, which remained intact. To identify infiltrating ECs, immunostaining was performed on slices of the Matrigel plug using an anti-mouse CD31 antibody.

### Aortic ring assay

The aortic ring assay was performed as previously described in Baker *et al*. (21). Briefly, aortic rings were isolated from 6-wk-old mice and cut to a fixed size in PBS. Growth factor–reduced Matrigel was spread into each well of a 96-well plate, which was incubated at 37°C for 20 min to allow matrix polymerization. Aortic rings were then placed on the matrices (1/well). Each embedded ring was cultured in Opti-MEM medium (31985070; Thermo Fisher Scientific) supplemented with 2.5% FBS and 50 ng/ml mouse VEGF-A. Medium was changed the following day and then every other day.

### Quantitative PCR

Quantitative PCR (qPCR; PikoReal; Thermo Fisher Scientific) was performed with Maxima Sybr Green/Rox qPCR Master Mix (K0221; Thermo Fisher Scientific) according to the manufacturer’s instructions. All results were normalized to levels of mouse glyceraldehyde-3-phosphate dehydrogenase (*Gapdh*). Primer sets for *Salm4*, prostaglandin-endoperoxide synthase 2 (*Ptgs2*), EC-specific molecule 1 (*Esm1*), collagen type VI α3 chain (*Col6a3*), and *Gapdh* are shown in **Table 1**.

**TABLE 1:** Primer sets used for RT-PCR and qPCR experiments

Primer	Sequence, 5′-3′	
	Forward	Reverse
Human primer sets for RT-PCR		
* SALM4*	CAGATCCAGTACAACAGCTCG	GCAGGTTCGGTGGAGAAG
* GAPDH*	AATCCCATCACCATCTTCCAG	TTCACACCCATGACGAACAT
Mouse primer sets for qPCR		
* Salm4*	CCGCATGTACCAGATCCAG	CACATAGATCATAGGTACGGCC
* Ptgs2*	AGGTCATTGGTGGAGAGGTG	CCTGCTTGAGTATGTCGCAC
* Esm1*	ACTGTCCCTATGGCACCTTC	CTCTTCTCTCACAGCGTTGC
* Col6a3*	GGTATCTCCGGGGAAGATGG	TCTCCAGAAGAACCAGGCAG
* Gapdh*	CTTTGTCAAGCTCATTTCCTGG	TCTTGCTCAGTGTCCTTGC

### mRNA sequencing and analysis

Total RNA was isolated using an RNeasy Kit (Qiagen, Germantown, MD, USA). The mRNA sequence libraries were prepared using the TruSeq Stranded mRNA Sample Prep Kit (RS-122; Illumina, San Diego, CA, USA). The protocols followed the TruSeq Stranded mRNA Sample Preparation Guide (Part 15031047 Rev. E). Sequencing was performed using an Illumina HiSeq 4000 sequencer (101-bp paired-end runs), the HiSeq 3000 4000 System User Guide Document 15066496 protocol, and TruSeq 3000 4000 Sequencing by Synthesis kit v.3 sequencing reagents. After removing low-quality and adapter sequences, sequence reads were aligned to the University of California–Santa Cruz, mouse genome reference sequence *Mus musculus* using Hierarchical Indexing for Spliced Alignment of Transcripts (HISAT) v.2.0.5 (22).

### Statistical analysis of gene expression

StringTie v.1.3.3b was used to estimate gene abundance (23). Abundance was measured in fragments per kilobase of exon per million fragments mapped; any values of 0 were discarded. To establish log_2_ transformation, 1 was added to each abundance value of filtered genes, and quantile normalization was performed. Statistical significance of differential expression data was determined using independent Student’s *t* tests. For the differentially expressed gene set, hierarchical clustering analysis was performed using complete linkage and Euclidean distance as a measure of similarity (absolute fold change, ≥2).

### *Trans*-endothelial/epithelial resistance assay

The permeabilities of HUVECs were assayed using Transwell chambers (3460; BD Biosciences) with polyester filters (0.4-μm pore size, 12-mm diameter). The upper wells were coated with 1% gelatin. HUVECs were trypsinized and resuspended in endothelial basal medium 2 containing endothelial growth medium 2 kit and 10% FBS to a final concentration of 8 × 10^4^ cells/ml. A 1-ml aliquot of cell suspension was added to each upper well and incubated at 37°C until cells formed junctions. Cells were then starved for 2 h in serum-free medium and treated with 50 ng/ml human VEGF-A for 30 min. Two electrodes were used for electrical measurements: one placed in the upper well and the other in the lower well, separated by the cellular monolayer.

### Miles vascular permeability assay

Six-to-eight–week-old mice were intravenously injected with PBS containing 100 μl 1% Evans blue (EB; E2129; MilliporeSigma). After 10 min, 50 μl mouse VEGF-A (50 ng/ml) or histamine (200 nmol/ml; H7125; MilliporeSigma) were intradermally injected into the skin of a shaved back. PBS and VEGF-A or histamine were injected into the dorsum of each mouse. Twenty-eight (4 groups of 7) mice were used in the assay. After 30 min, the back skin was biopsied using a 6-mm punch and then incubated in formamide solution overnight at 56°C to extract the dye. The absorbance of the supernatant was measured on a spectrophotometer at 620 nm. The dye content in the skin was calculated against a standard curve and expressed as μg/g.

### I/R model through middle cerebral artery occlusion

Eight-to-ten–week-old mice were anesthetized by intraperitoneal injection of tribromoethanol (Avertin, 2.5%). A heating pad (JD-OT-03106; Jeung-Do Bio and Plant, Seoul, South Korea) was used to maintain a body temperature of 37°C during surgery. I/R was induced by middle cerebral artery occlusion on the left side as previously described in Zhang *et al*. (24). Briefly, the left common carotid artery (CCA) was exposed and separated from the vagus nerve. The left CCA was ligated at the distal side of its intersection. The left external carotid artery was dissected free and ligated at the distal side of the CCA intersection. A 6-0 silicone rubber-coated monofilament suture (602156PK10; Doccol, Sharon, MA, USA) was inserted into the left external carotid artery stump and gently advanced 8 mm into the internal carotid artery. The suture proceeded from the internal carotid artery to the middle cerebral artery to occlude the middle cerebral artery. After 3 h of ischemia, reperfusion was produced by withdrawal of the suture. In the sham-treated group, the same surgical procedures were performed without insertion of the suture.

### Neurologic scores

Neurologic defects were evaluated after 16 h reperfusion as previously described in Zhang *et al*. (24). The scores were as follows: 0, no observable neurologic deficit; 1, flexion of contralateral torso and forelimbs upon lifting the whole animal by the tail; 2, circling to the contralateral side when held by the tail with feet on the floor; 3, spontaneous circling to the contralateral side; 4, no spontaneous motor activity. For consistency, scoring was repeated 3 times.

### Measurement of infarction volume

Infarction volume was measured as previously described in Zhang *et al*. (24). Briefly, after 16 h reperfusion, mice were anesthetized by intraperitoneal injection of tribromoethanol (Avertin, 2.5%). Brains were harvested and cut into 2 mm coronal slices using a brain matrix. Each slice was incubated with 2% 2,3,5-triphenyltetrazolium chloride (TTC) at 37°C for 20 min. After the TTC reaction, infarction was marked by unstained areas, whereas normal areas were stained red. Infarction was quantified using ImageJ software. To eliminate the effects of edema in the ipsilateral hemisphere, the infarction volume was calculated as follows: [(contralateral hemisphere − undamaged ipsilateral hemisphere) ÷ (contralateral hemisphere × 2) × 100] %.

### Determination of blood-brain barrier permeability and brain-water content

Blood-brain barrier (BBB) permeability was assessed by EB extravasation using a method previously described in Zhang *et al*. (24) with slight modification. Briefly, after 16 h reperfusion, EB (2% in 0.9% saline, 4 ml/kg) was injected into the tail vein. Following 3 h after injection, mice were anesthetized by intraperitoneal injection of tribromoethanol (Avertin, 2.5%). Brains were harvested, and each hemisphere was weighed, homogenized in PBS, and centrifuged at 2000 *g* for 15 min at 4°C. The resulting supernatant was added to an equal volume of trichloroacetic acid. After overnight incubation and centrifugation at 2000 *g* for 15 min at 4°C, the absorbance of the supernatant was measured by spectrophotometer at 620 nm. The dye content in the brain was calculated against a standard curve and expressed as micrograms/gram. Brain-water content was calculated using the following formula: [(wet weight − dry weight) ÷ wet weight × 100] %. After 16 h reperfusion, ipsilateral and contralateral hemispheres were weighed before (wet weight) and after drying at 80°C overnight (dry weight).

### Animal studies

All animal facilities and experiments were approved by the Animal Core Facility at Yonsei University College of Medicine and the Yonsei Laboratory Animal Research Center at Yonsei University.

### Statistical analysis

Data are presented as means ± sd or sem. Multiple group means were compared by 1-way ANOVA with Dunnett’s or Turkey’s corrections, followed by the 2-tailed Student’s *t* test for pairwise comparisons.

## RESULTS

### SALM4 is expressed in ECs

**Figure 1:**
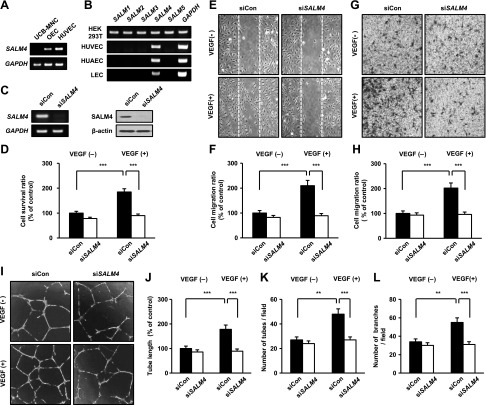
SALM4 is required for HUVEC survival, migration, and tube formation. *A*) Expression of *SALM4* mRNA during differentiation from UCB-MNCs to OECs and then HUVECs. *B*) *SALMs* mRNA expression in human embryonic kidney 293 transformed (HEK 293T) cell lines, HUVECs, human umbilical artery ECs (HUAECs), and lymphatic ECs (LECs). *C*) mRNA and protein expression were measured by RT-PCR and Western blot analysis in HUVECs transfected with siCon or siSALM4 (80 nM, 48 h). *D*) HUVEC survival was measured by MTT assay. *E*, *F*) Migration of SALM4-knockdown HUVECs on gelatin was photographed at 8 h (*E*) and quantified (*F*). *G*, *H*) Chemotactic motility was evaluated using the Boyden chamber assay on gelatin (*G*) and quantified (*H*). *I*) Tube structure formation on Matrigel in SALM4-silenced HUVECs was photographed at 10 h. *J*–*L*) Total tube length (*J*), number of tubes (*K*), and number of branches/field (*L*) were quantified. VEGF-A was used at 40 ng/ml. Error bars represent means ± sd. **P < 0.01, ***P < 0.001 by paired, 2-tailed Student’s *t* test.

We previously performed Affymetrix gene chip analysis of UCB-MNCs, which were characterized as hematopoietic monocytes, during their differentiation into outgrowth ECs (OECs) (Gene Expression Omnibus accession GSE12891) (25). The analysis revealed that *SALM4* was highly expressed in the OEC stage compared with the UCB-MNC stage, which was confirmed by RT-PCR (**Fig. 1*A***). We also examined the expression of other SALM family members in HUVECs, human umbilical artery ECs, and lymphatic ECs. *SALM4* was expressed in all 3 cell types. Human embryonic kidney 293 transformed cell lines were used as a positive control for *SALM1−5* expression (Fig. 1*B*). To determine the expression of *Salm4* in mouse ECs, we examined *Salm4* expression in organs of *Salm4^−/−^* mice. Frozen mouse organs were sectioned and treated with DAPI as a nuclear marker. Tissues were immunostained for CD31 as an EC marker and β-galactosidase for *Salm4* expression. *Salm4* was expressed in the ECs of the brain, liver, and lungs (Supplemental Fig. S1), suggesting potential functions in ECs.

### SALM4 regulates EC survival, migration, and capillary-like tube formation

To investigate whether SALM4 has important roles in endothelial function, we used 3 types of siSALM4. SALM4 mRNA and protein expression levels were inhibited in siSALM4-transfected HUVECs (Fig. 1*C* and Supplemental Fig. S2*A*–*C*). We then evaluated the effects of SALM4 knockdown on EC properties. In a previous study, when SALM4 was overexpressed, knockdown of flotillin-1 prevented neurite branching by SALM4 (13). Thus, in the present study, we examined this function in ECs. Cell viability was evaluated by MTT assay and cell counting. siSALM4 decreased the viability of HUVECs treated with VEGF-A (Fig. 1*D* and Supplemental Fig. S2*D*). Knockdown of SALM4 also inhibited wound-healing migration in the presence of VEGF-A stimulation (Fig. 1*E*, *F*). Reduced migration was observed with 3 types of siSALM4-transfected HUVECs, indicating that this function is not an off-target effect of siRNA (Supplemental Fig. S2*E*, *F*). Chemotactic motility was inhibited in SALM4-depleted HUVECs upon VEGF-A stimulation (Fig. 1*G*, *H*). Furthermore, a well-organized tube-like formation was observed in siCon-transfected HUVECs, but it was severely impaired in SALM4-silenced HUVECs with VEGF-A treatment (Fig. 1*I–L*). These results indicate that SALM4 can modulate VEGF-A–induced angiogenic functions in ECs.

### VEGF-A–induced angiogenic sprouting is inhibited in *Salm4^−/−^* mice

**Figure 2:**
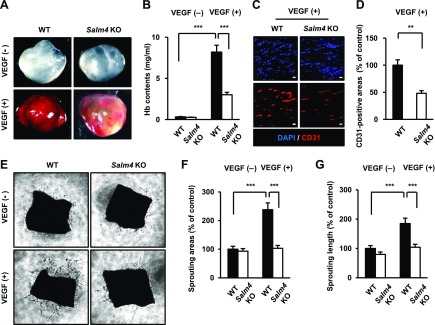
*Salm4^−/−^* mice show reduced angiogenic sprouting upon VEGF-A treatment. *A*) Matrigel plugs were implanted into 6-wk-old WT and *Salm4^−/−^* mice. Matrigel was mixed with PBS [VEGF-A (−) group] or 200 ng/ml VEGF-A [VEGF-A (+) group]; *n* = 7/group. Images show Matrigel plugs at d 7 after implantation. *B*) Quantification of hemoglobin (Hb) from Matrigel plugs from WT and *Salm4^−/−^* mice. *C*, *D*) Immunostaining of Matrigel sections: nucleus, DAPI; blood vessels, CD31 (*C*); quantification of relative CD31-positive areas (*D*). Scale bars, 20 μm. *E*) Aortic ring assay of 6-wk-old WT and *Salm4^−/−^* mice. VEGF-A was used at 50 ng/ml (*n* = 7/group). *F*, *G*) Quantification of relative sprouting areas (*F*) and filopodial length (*G*). Ns, not significant. Error bars represent means ± sd. **P < 0.01, ***P < 0.001 by paired, 2-tailed Student’s *t* test.

We analyzed the phenotype of *Salm4^−/−^* mice to examine the angiogenic effects of SALM4 deficiency *in vivo*. Body weight, fertility, development, and organ vasculature were not affected by *Salm4* KO (Supplemental Fig. S3*A*–*F*). We then performed an *in vivo* Matrigel plug assay. Before performing this experiment, we confirmed that *Salm4* KO did not alter vessel density in the skin (Supplemental Fig. S4*A*, *B*). In the VEGF-A (−) group, no significant difference was observed in Matrigel plugs from WT and *Salm4^−/−^* mice. In the VEGF-A (+) group, VEGF-A–induced neovascularization was inhibited in plugs from *Salm4^−/−^* mice compared with WT mice (**Fig. 2*A***). This finding was confirmed by hemoglobin contents (Fig. 2*B*). In addition, fewer CD31-positive cells were observed in confocal images of plugs from *Salm4^−/−^* mice compared with WT mice (Fig. 2*C*, *D*). To determine whether the phenotype of *Salm4^−/−^* mice is VEGF-A dependent, we examined FGF-2–induced neovascularization. Similar to VEGF-A, FGF-2 induces survival, proliferation, and migration of ECs (26, 27). In the FGF-2 (−) and FGF-2 (+) groups, no significant differences were observed in the Matrigel plugs from WT and *Salm4^−/−^* mice (Supplemental Fig. S4*C*–*F*). We further examined the sprouting activity of ECs in *Salm4^−/−^* mice using an *ex vivo* aortic ring assay. In the VEGF-A (−) group, no significant differences were observed in aortic rings from WT and *Salm4^−/−^* mice. In the VEGF-A (+) group, EC sprouting areas and length were inhibited in aortic rings of *Salm4^−/−^* mice compared with those of WT mice (Fig. 2*E–G*). Taken together, these results suggest that loss of SALM4 inhibits angiogenesis in response to VEGF-A.

### VEGF-A–induced vascular permeability is attenuated in SALM4-knockdown HUVECs and *Salm4^−/−^* mice

**Figure 3:**
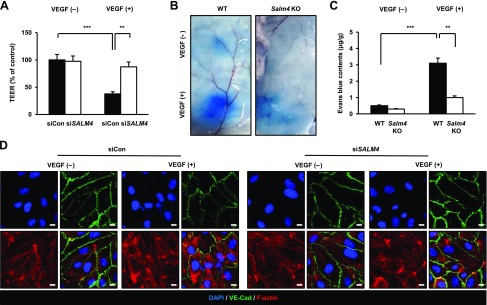
VEGF-A–induced permeability is inhibited in SALM4-depleted HUVECs and *Salm4^−/−^* mice. *A*) Permeability was measured by the *trans*-endothelial/epithelial resistance (TEER) assay. VEGF-A at 50 ng/ml was used for siCon- and siSALM4-transfected HUVECs. *B*) EB leakage was assessed 30 min after intradermal injection of PBS [VEGF-A (−) group] or 50 ng/ml VEGF-A [VEGF-A (+) group]. Six-week-old mice were used, *n* = 7/group. *C*) EB quantification was measured by absorbance at 620 nm. *D*) siCon- and siSALM4-transfected HUVECs were stained with DAPI, VE-cadherin (VE-Cad), and F-actin. Ns, not significant. Error bars represent means ± sd. Scale bars, 10 μm. **P < 0.01, ***P < 0.001 by paired, 2-tailed Student’s *t* test.

VEGF-A is a vascular permeability factor that plays an essential role in physiologic and pathologic angiogenesis (28). As shown by the *in vitro* *trans*-endothelial/epithelial resistance assay, VEGF-A markedly induced vascular permeability in the siCon-transfected HUVECs. By contrast, siSALM4-transfected HUVECs did not exhibit VEGF-A–induced permeability (**Fig. 3*A***). To identify whether this effect also occurred *in vivo*, the Miles vascular permeability assay was performed. In the VEGF-A (−) group, no significant difference was observed in skin from WT and *Salm4^−/−^* mice. In the VEGF-A (+) group, VEGF-A strongly induced vascular permeability in WT mouse skin, as shown by increased EB leakage. *Salm4^−/−^* mouse skin showed reduced leakage of EB compared with WT mouse skin (Fig. 3*B*). Reduced extravasation of EB in *Salm4^−/−^* mouse skin was also confirmed by quantification of EB contents (Fig. 3*C*). Because histamine also induces vascular permeability (29), we examined whether *Salm4* KO affects histamine-induced permeability. In the histamine (−) and histamine (+) groups, no significant differences were observed in the skin from the WT and *Salm4^−/−^* mice (Supplemental Fig. S4*G*, *H*).

VEGF-A disrupts junction proteins and alters actin filament distribution (30). Therefore, we assessed these effects in SALM4-knockdown HUVECs. The VEGF-A–untreated group retained the polygonal shape and linear pattern of VE-cadherin at cell borders, confirming junction formation. VEGF-A–treated HUVECs transfected with siCon showed disrupted junctions and breakage of the linear VE-cadherin pattern. However, knockdown of SALM4 prevented these effects. Furthermore, VEGF-A–induced stress fiber formation was increased, and cortical actin ring structure was decreased in control HUVECs using F-actin staining. By contrast, reduced VEGF-A–induced stress fiber formation and increased cortical actin ring structure were observed in the absence of SALM4 (Fig. 3*D*). Collectively, these results indicate that depletion of SALM4 inhibits EC permeability in response to VEGF-A.

### SALM4 controls VEGFR2-Y1175 phosphorylation in response to VEGF-A stimulation

**Figure 4:**
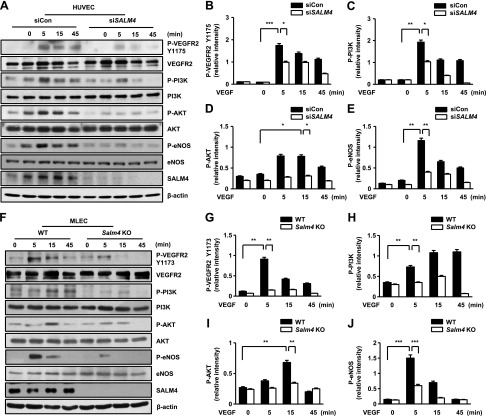
SALM4 regulates VEGFR2 phosphorylation at Y1175 (Y1173 in mice) and downstream signaling in HUVECs and MLECs stimulated with VEGF-A. *A*) Effects of SALM4 knockdown on signaling pathways induced by VEGF-A (20 ng/ml) in HUVECs. *B–E*) Blots were assessed using ImageJ software; *n* = 3 independent experiments. *F*) Effects of *Salm4* KO on signaling pathways induced by VEGF-A (20 ng/ml) in MLECs. *G–J*) Quantification of blots using ImageJ software; *n* = 3 independent experiments. Error bars represent means ± sd. Blots incubated with phospho-specific antibodies were probed only once from the different gels. Other blots were stripped and reprobed. P-, phosphorylated. *P < 0.05, **P < 0.01, ***P < 0.001 by paired, 2-tailed Student’s *t* test.

VEGF-A–induced angiogenic functions, such as migration, survival, differentiation, proliferation, and vascular tube formation, were mainly affected by VEGFR signaling (15). VEGF-A binding to VEGFR2 and NRP1 lead to a conformational change of the VEGFR2 autophosphorylation site, triggering the activation of downstream kinases (15, 31). To determine the mechanism responsible for the impaired VEGF-A response, we transfected siCon or siSALM4 into HUVECs stimulated with VEGF-A. We examined the major phosphorylation sites of VEGFR2 (human tyrosine residues Y951, Y1175, and Y1214; mouse tyrosine residues Y949, Y1173, and Y1212). VEGF-A–induced VEGFR2 phosphorylation at Y1175 was enhanced in control HUVECs but not in SALM4-silenced HUVECs (**Fig. 4*A*, *B***). VEGFR2 phosphorylation at Y1175 activates ERK and PI3K-AKT-eNOS and regulates migration, survival, and permeability (15). Activation of PI3K-AKT-eNOS was reduced, but ERK was unchanged in SALM4-silenced HUVECs compared with siCon-transfected HUVECs (Fig. 4*A–E* and Supplemental Fig. S5*A*, *B*). VEGF-A–treated WT MLECs also showed enhanced activation of VEGFR2 phosphorylation at Y1173 and activation of PI3K-AKT-eNOS. VEGFR2 phosphorylation at Y1173 was reduced in *Salm4^−/−^* MLECs compared with WT MLECs. Activation of PI3K-AKT-eNOS was reduced, but ERK was unchanged in *Salm4^−/−^* MLECs compared with WT MLECs (Fig. 4*F–J* and Supplemental Fig. S5*C*, *D*). Other VEGFR2 phosphorylation sites, namely, Y951 and Y1214, were also unchanged in SALM4-knockdown HUVECs compared with siCon-transfected HUVECs (Supplemental Fig. S5*E*–*G*). These observations were confirmed in *Salm4^−/−^* MLECs (Supplemental Fig. S5*H*–*J*).

Because VEGFR2 phosphorylation is regulated by binding to the coreceptor and PTP, we examined whether SALM4 could bind to these molecules (16). NRPs (NRP1 and NRP2) bind VEGF family ligands, acting as coreceptors along with the 3 receptor tyrosine kinases, VEGFR1, VEGFR2, and VEGFR3. When VEGF-A binds to NRPs and to VEGFR1 or VEGFR2 in ECs, the resulting complex undergoes conformational changes (32). SALM4 did not bind to NRP1, NRP2, VEGFR1, or VEGFR2 (Supplemental Fig. S6*A*–*F*). VEGF-A–VEGFR1-NRP1 or NRP2 and VEGF-A–VEGFR2-NRP1 or NRP2 were immunoprecipitated from SALM4-silenced HUVECs stimulated with VEGF-A (Supplemental Fig. S6*A*–*D*). PTP1B and VE-PTP directly detach phosphorus from Y1175 of VEGFR2 (33, 34). SALM4 did not bind to PTP1B and VE-PTP (Supplemental Fig. S6*G*). Therefore, SALM4 does not directly regulate VEGFR2 phosphorylation through these mechanisms. Taken together, these results demonstrate that SALM4 knockdown inhibits angiogenic functions through VEGFR2 phosphorylation at Y1175 and not Y951 or Y1214.

### Angiogenesis-related gene transcription is inhibited in *Salm4^−/−^* MLECs stimulated with VEGF-A

To investigate transcriptional changes elicited by VEGFR2 activation, we performed mRNA sequencing analysis of isolated lung ECs from WT and *Salm4^−/−^* mice. MLECs were immunostained for VE-cadherin to confirm these were ECs before performing analysis (Supplemental Fig. S7*A*). Because well-known VEGF-A–VEGFR2 signaling target genes, such as regulator of calcineurin 1, ESM1, and angiopoietin 2, are up-regulated after 4 or 8 h of VEGF-A treatment, we stimulated with VEGF-A in MLECs at 0, 4, and 8 h (35). We identified 23 significantly up-regulated genes at 4 h and 10 significantly up-regulated genes at 8 h in WT MLECs (absolute fold change, >2; *P* < 0.01). Because these genes were not induced in *Salm4^−/−^* MLECs (Supplemental Fig. S7*B*), we proposed that these 33 genes are targets of VEGFR2 phosphorylation at Y1173. Consistent with previous reports, ESM1 (36) and PTGS2 (37), which is positively regulated downstream of VEGF-A–VEGFR2 signaling, were induced by VEGF-A treatment at 4 h in WT MLECs but not in *Salm4^−/−^* MLECs (Supplemental Fig. S7*B*, *C*). COL6A3, which is positively regulated downstream of PI3K-AKT signaling in ECs (38), was induced at 8 h in WT MLECs but not in *Salm4^−/−^* MLECs (Supplemental Fig. S7*B*, *C*). These results suggest that *Salm4* KO inhibits angiogenic gene transcription induced by VEGFR2-Y1173 phosphorylation.

### *Salm4^−/−^* mice show ameliorated brain damage after I/R

**Figure 5:**
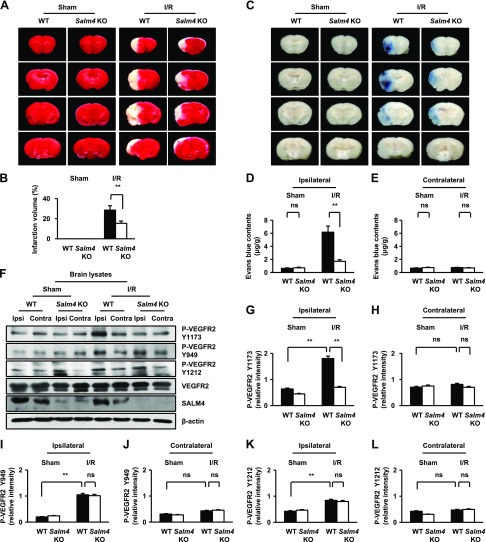
*Salm4^−/−^* mice exhibit attenuated brain damage and VEGFR2 phosphorylation (P-VEGFR2) at Y1173 after I/R. *A*, *B*) Comparison of infarction volume by TTC staining in the sham-treated and I/R groups from WT and *Salm4^−/−^* mice (*A*) and quantification of infarction volume (*B*). *C*–*E*) EB extravasation from sham-treated and I/R groups from WT and *Salm4^−/−^* mice (*C*) and quantification of EB in ipsilateral (*D*) and contralateral hemispheres (*E*); *n* = 7/group. *F*) Effects of *Salm4* KO on VEGFR2 signaling in brain lysates. *G*–*L*) Quantification of blots using ImageJ software; Y1173 (*G*, *H*), Y949 (*I*, *J*), Y1212 (*K*, *L*); *n* = 3 independent experiments. Contra, contralateral hemisphere; ipsi, ipsilateral hemisphere; ns, not significant. Error bars represent means ± sd. **P < 0.01 by paired, 2-tailed Student’s *t* test.

As mentioned above, SALM4-depleted ECs showed attenuated permeability through VEGFR2 phosphorylation at the Y1175-PI3K-AKT-eNOS signaling axis, which plays a role in acute brain stroke (39). Acute stroke increases vascular permeability, and leaky blood vessels aggravate BBB disruption and edema (40). Thus, we used the I/R model to identify SALM4 functions in a pathologic condition (Supplemental Fig. S8*A*). In the sham-treated group, no neurologic deficits were observed 16 h after reperfusion in WT and *Salm4^−/−^* mice. In the I/R group, severe neurologic deficits were present in WT mice but not *Salm4^−/−^* mice (Supplemental Fig. S8*B*). In the sham-treated group, TTC staining indicated that there were no areas of infarction in WT or *Salm4^−/−^* mice. In the I/R group, there were fewer infarcted regions in *Salm4^−/−^* mice compared with WT mice (**Fig. 5*A*, *B***). No significant differences in BBB permeability, assessed by EB extravasation, were observed in the sham-treated WT and *Salm4^−/−^* mice. In the I/R group, EB extravasation was observed in WT mice. However, *Salm4^−/−^* mice did not show ischemia-induced EB extravasation (Fig. 5*C–E*). Next, we measured water content 16 h after reperfusion by the wet-dry method. In the sham-treated group, no significant differences were observed in WT and *Salm4^−/−^* mice. In the I/R group, the brain-water content was increased in WT mice compared with *Salm4^−/−^* mice (Supplemental Fig. S8*C*, *D*). To identify which effects of SALM4 on VEGFR2 phosphorylation are due to I/R, we examined VEGFR2 phosphorylation sites in brain lysates. In the sham-treated group, no significant differences were observed in WT and *Salm4^−/−^* brains. In the I/R group, VEGFR2 phosphorylation was induced at Y949, Y1173, and Y1212 in WT brains. However, *Salm4^−/−^* brains showed reduced VEGFR2 phosphorylation only at Y1173 (Fig. 5*F–L*). Therefore, *Salm4^−/−^* brains can ameliorate I/R damage through inhibition of VEGFR2-Y1173 in ECs.

### *Salm4^−/−^* mice show attenuated BBB disruption after I/R

**Figure 6:**
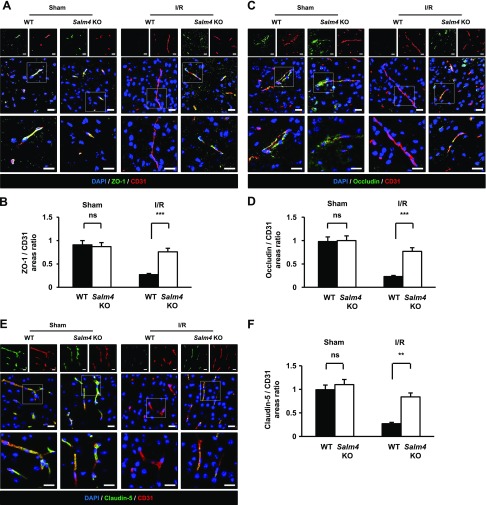
Brains of *Salm4^−/−^* mice prevent disruption of tight junction-related proteins after I/R. *A*) Immunostaining for ZO-1 and CD31 in ischemic brain sections of sham-treated and I/R groups. Merged images of ZO-1 and CD31 staining are also shown. Square: enlarged image of the region. *B*) Quantification of ZO-1– positive blood vessels. *C*) Immunostaining for occludin and CD31 in ischemic brain sections. Merged images of occludin and CD31 staining are shown. Square: enlarged image of the region. *D*) Quantification of occludin-positive blood vessels. *E*) Immunostaining for claudin-5 and CD31 in ischemic brain sections. Merged images of claudin-5 and CD31 staining are shown. Square: enlarged image of the region. Scale bars, 20 μm. *F*) Quantification of claudin-5-positive blood vessels; *n* = 5/group. Ns, not significant. Error bars represent means ± sd. **P < 0.01, ***P < 0.001 by paired, 2-tailed Student’s *t* test.

The tight junction-related proteins ZO-1, occludin, and claudin-5 were examined 16 h after reperfusion by conjunction with CD31, an EC marker. In the sham-treated group, ZO-1 and CD31 were nearly merged. In the I/R group, WT brains showed greatly reduced ZO-1 expression, indicative of a disrupted BBB, compared with ZO-1 of *Salm4^−/−^* brains. A superimposed lining of ZO-1 and CD31 was observed in *Salm4^−/−^* brains in the I/R group, suggesting that BBB destruction was attenuated (**Fig. 6*A*, *B***). Similar results were observed for occludin and claudin-5 (Fig. 6*C–F*). These results further demonstrate that *Salm4^−/−^* brains exhibit attenuated disruption of the BBB, potentially through inhibition of VEGFR2-Y1173 in ECs.

### *Salm4^−/−^* mice show suppressed expression of adhesion molecules and activation of glial cells after I/R

**Figure 7:**
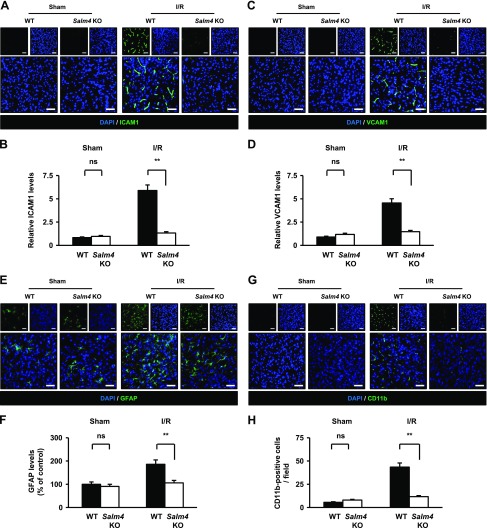
Brains of *Salm4^−/−^* mice showed attenuated expression of adhesion molecules and activation of glial cells after I/R. *A*, *B*) Immunostaining for ICAM1 in ischemic brain sections of sham-treated and I/R groups (*A*) and quantification of relative ICAM1 levels (*B*). *C*, *D*) Immunostaining for VCAM1 in ischemic brain sections (*C*) and quantification of relative VCAM1 levels (*D*). *E*, *F*) Immunostaining for GFAP in ischemic brain sections (*E*) and quantification of GFAP expression levels (*F*). *G*, *H*) Immunostaining for CD11b in ischemic brain sections (*G*) and quantification of CD11b-positive cells (*H*). Ns, not significant. Error bars represent means ± sd; *n* = 5/group. Scale bars, 50 μm. **P < 0.01 by paired, 2-tailed Student’s *t* test.

The neuroinflammatory response may be an important factor in VEGF-A–mediated BBB disruption during brain ischemic stroke (41, 42). In the sham-treated group, ICAM1 and VCAM1 were barely expressed in WT and *Salm4^−/−^* brains. In the I/R group, ICAM1 and VCAM1 were significantly decreased in *Salm4^−/−^* brains compared with WT brains 16 h after reperfusion (**Fig. 7*A–D***).

Activation of glial cells, such as astrocytes and microglia, are induced by cerebral ischemia (43, 44). Based on this report, we investigated the number of astrocytes and microglia in the I/R model. In the sham-treated group, no significant changes were observed in WT and *Salm4^−/−^* brains. In the I/R group, WT brains showed activation of GFAP-positive astrocytes and CD11b-positive microglia. However, *Salm4^−/−^* brains showed significantly reduced activation of astrocytes and microglia 16 h after reperfusion (Fig. 7*E–H*). Taken together, *Salm4^−/−^* brains suppress neuroinflammation, presumably through inhibition of VEGFR2-Y1173 in ECs.

## DISCUSSION

This study identified SALM4 as a novel regulator of VEGF-A–induced VEGFR2-Y1175 phosphorylation and associated angiogenic functions, including migration, tube-like formation, EC recruitment, and vascular permeability (Supplemental Fig. S9). It also established SALM4 as a fine controller of brain ischemic reperfusion injury.

VEGFR2 signaling should be precisely regulated, because aberrant VEGFR2 phosphorylation causes abnormal EC sprouting and permeability (1). Based on these results, *in vivo* studies of VEGFR2 phosphorylation sites were performed. VEGFR2-Y949F and Y1212F knock-in mice are viable and fertile (16). However, Li *et al.* (45) reported that VEGFR2-Y949F knock-in mice demonstrate reduced glioblastoma permeability, B16F10 tumor vascular leakage, and metastasis. VEGFR2-Y1173F knock-in mice show embryonic lethality between 8.5 and 9.5 d, similar to the phenotypes of *Vegfr2* and *Vegf-a* KO mice. These results suggest that Y1175 has essential roles in VEGFR2 activation (46). The present study showed that *Salm4^−/−^* mice have attenuated VEGFR2 phosphorylation at Y1173, but not at Y949 or Y1212, following brain I/R injury. This finding indicates that SALM4-mediated potentiation of VEGFR2 phosphorylation at Y1173 plays critical roles in neurologic impairments, BBB disruption, and neuroinflammation following I/R damage.

We used several strategies to delineate the mechanism of SALM4-mediated VEGFR2 signaling. The strength of VEGFR2 signaling is tightly regulated at numerous levels, including by PTPs, coreceptors, and receptor internalization (16). There are 2 categories of PTPs that dephosphorylate VEGFR2: receptor type PTPs, such as VE-PTP and PTP receptor type J, and intracellular PTPs, such as PTP1B, T-cell PTP, Src homology 2 domain–containing proteins 1 and 2, PTP-non-receptor type 9, and low MW–PTP (44). Among these, VE-PTP and PTP1B are well characterized. The *Ve-ptp* KO mouse phenotype is embryonic lethal at 9.5 d, suggesting that VE-PTP is required for endothelial development (33, 34). VE-PTP dephosphorylates all tyrosine residues of VEGFR2 (47). PTP1B dephosphorylates VEGFR2 at Y1175 and decreases ERK activation in early endosomes (48). VE-PTP and PTP1B bind to VEGFR2 and elicit conformational changes that detach phosphorus from VEGFR2 (16). Because SALM4 did not bind to VEGFR2, we examined whether SALM4 could bind to PTPs. However, we did not find evidence that SALM4 binds directly to VE-PTP or PTP1B. Therefore, SALM4 does not seem to affect recruitment of PTPs. Next, NRP1, an indispensable coreceptor of VEGFR2, binds to VEGFR2 only in the presence of VEGF-A stimulation (49). SALM4-depleted ECs formed a VEGF-A–NRP1-VEGFR2 complex as siCon-transfected ECs. Because SALM4 did not bind to NRP1, SALM4 does not regulate VEGFR2 phosphorylation through this mechanism. Similar to SALM4, Zhang *et al.* (50) reported that roundabout guidance receptor 4 also inhibits VEGFR2 phosphorylation. Roundabout guidance receptor 4 binds UNC5B, and the UPA domain of UNC5B reduces VEGFR2 phosphorylation at Y951 but not at Y1175. These possibilities suggest that SALM4 regulates the folding of VEGFR2-Y1175 by binding to an unidentified molecule. Another possibility is that VEGFR2 signaling is regulated by receptor internalization. The VEGF-A–NRP1-VEGFR2 complex is internalized and binds to synectin–myosin-VI. The internalized VEGFR2 complex then undergoes small GTPase of the Ras superfamily 11(*Rab*11)dependent recycling. Because internalized VEGFR2 is phosphorylated at Y951 and Y1175, the downstream kinases SRC and ERK are phosphorylated, respectively (51). SALM4 depletion in ECs did not alter ERK phosphorylation compared with siCon-transfected ECs, suggesting that SALM4 is unlikely to affect VEGFR2 internalization. SALM4 was previously reported to regulate neurite branching through interactions with lipid raft–associated proteins (13). In the present study, we demonstrated the vascular role of SALM4 in VEGFR2 signaling in ECs. Thus, SALM4 is likely to act as a modulator of receptor-mediated signal transduction in the membranes of neuronal cells and ECs. Although we could not identify the exact interacting protein and mechanism by which SALM4 regulates VEGFR2 phosphorylation, its action in ECs appears specific to VEGFR2-Y1175 and not Y951 or Y1214.

Reduced VEGF-A–VEGFR2-Y1175–PI3K-AKT-eNOS signaling in SALM4 inhibited ECs, suggesting that VEGF-A–induced disruption of endothelial junction is reduced in SALM4-silenced ECs. Such signaling is important for regulating vascular permeability. Acute brain I/R damage can increase VEGF-A expression in neurons, astrocytes, macrophages, and ECs. Increased VEGF-A influences vascular permeability through PI3K-AKT phosphorylation, which activates eNOS (52–54). Phosphorylation of VEGFR2, a major receptor for VEGF-A, leads to degradation of endothelial junction molecules after stroke (55). Consequently, ischemic brains have vascular leakage, causing the infiltration of inflammatory molecules into the cerebral blood flow. Behavioral defects, areas of infarction, albumin extravasation, edema, BBB disruption, neuroinflammation, and glial cell activation were reduced after I/R in the brains of *Salm4^−/−^* mice. Furthermore, VEGFR2 phosphorylation at Y949, Y1173, and Y1212 was induced in WT brains after I/R, but only VEGFR2 phosphorylation at Y1173 was reduced in *Salm4^−/−^* brains. The current treatment for acute ischemia is largely dependent on tissue plasminogen activator–mediated thrombolysis. However, this treatment exacerbates the risk of hemorrhage, which is related to BBB breakdown (56). Because BBB stabilization increases the efficacy of tissue plasminogen activator treatment, BBB stabilization is critical for reducing poor outcomes in patients with acute ischemia. Based on our findings, the VEGFR2-Y1175–PI3K-AKT-eNOS signaling axis is a potentially novel therapeutic target for vascular permeability–related diseases, such as brain ischemic reperfusion and tumor progression.

In summary, we suggest that SALM4 induces conformational changes in the proximity of VEGFR2 phosphorylation at Y1175 by binding an unidentified mediator in the lipid raft, which is the platform for signaling machinery in the plasma membrane (57). Future research is needed to fully elucidate the role of SALM4 in VEGFR2 phosphorylation at Y1175.

## Supplementary Material

This article includes supplemental data. Please visit *http://www.fasebj.org* to obtain this information.

## Abbreviations

AKT: protein kinase B
BBB: blood-brain barrier
CCA: common carotid artery
Col6a3: collagen type VI α3
EB: Evans blue
EC: endothelial cell
ESM1: EC-specific molecule 1
F-actin: fibrous actin
FBS: fetal bovine serum
FGF: fibroblast growth factor
FLRT2: fibronectin leucine-rich transmembrane protein 2
Gapdh: glyceraldehyde-3-phosphate dehydrogenase
GFAP: glial fibrillary acidic protein
I/R: ischemia and reperfusion
ICAM: intercellular adhesive molecule
KO: knockout
LRR: leucine-rich repeat
MLEC: mouse lung endothelial cell
MTT: 3-(4,5-dimethyl-2-thiazolyl)-2,5-diphenyl-2H-tetrazolium bromide
NRP: neuropilin
OCT: optimal cutting temperature
OEC: outgrowth EC
Ptgs2: prostaglandin-endoperoxide synthase 2
PTP: protein tyrosine phosphatase
qPCR: quantitative PCR
SALM: synaptic adhesion-like molecule
siCon: control siRNA
siRNA: small interfering RNA
siSALM4: SALM4 siRNA
TTC: 2,3,5-triphenyltetrazolium chloride
UCB-MNC: umbilical cord blood mononuclear cell
UNC5B: UNC-5 Netrin Receptor B
VCAM1: vascular cell adhesion molecule 1
VE: vascular endothelial
VEGFR: VEGF receptor
WT: wild type
ZO-1: zona occludens-1
