# Supplementary material for: SALM4 regulates angiogenic functions in endothelial cells through VEGFR2 phosphorylation at Tyr1175
Source: FASEB J. 2019 Jun 6;33(9):9842–57. doi: 10.1096/fj.201802516RR (PMC6704462; doi:10.1096/fj.201802516RR)
Supplement: Supplementary file 1 [file fj.201802516RR.sf1.docx]

**Supplemental Figures**

**
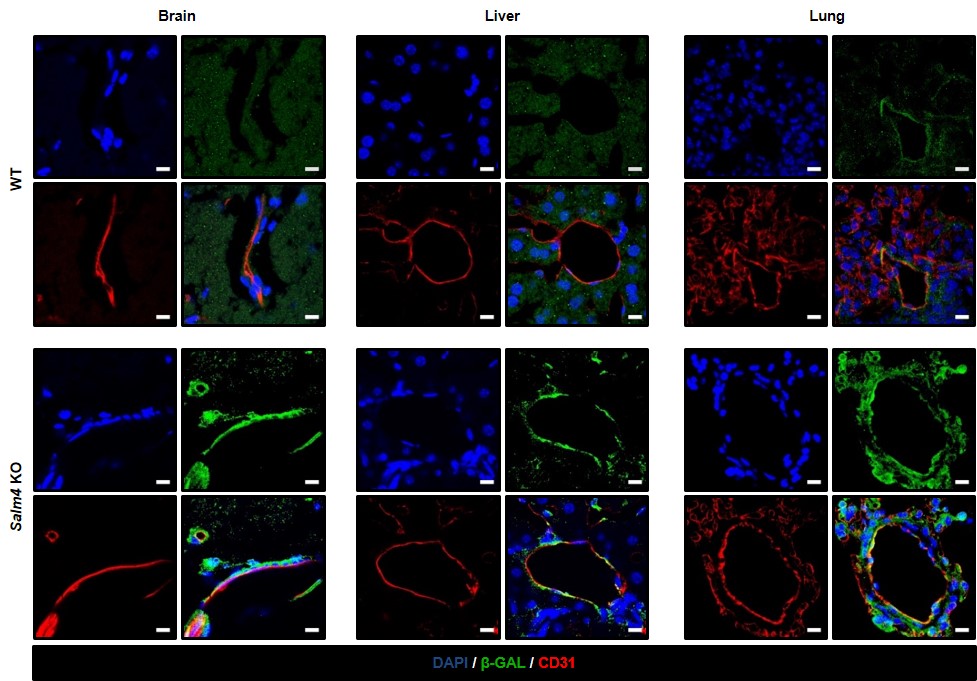
**

**Figure S1. SALM4 is expressed in endothelial cells.**

Immunostaining of brain, liver, and lung sections from 6-week-old WT and *Salm4^−/−^* mice: nucleus, DAPI; SALM4, β-GAL; blood vessels, CD31. Scale bars: 10 μm.

**
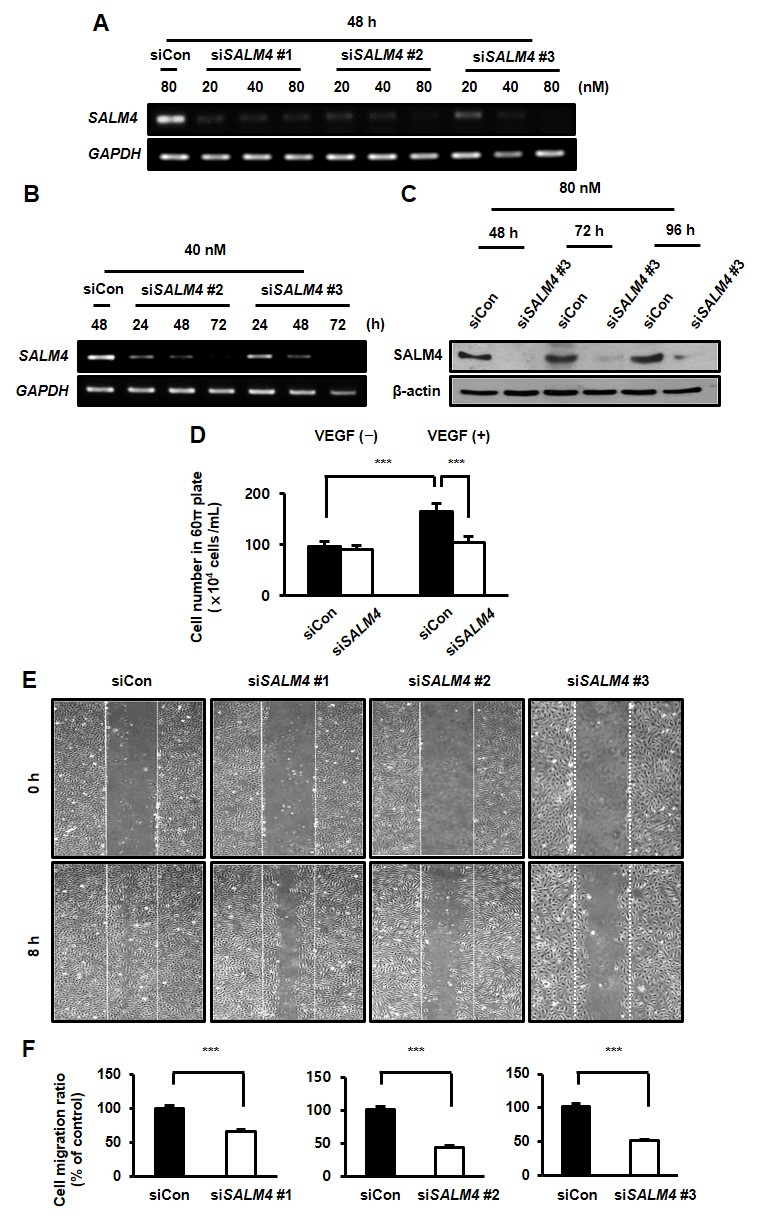
**

**Figure S2. SALM4 knockdown inhibits VEGF-A-induced survival and inhibits migration of HUVECs.**

**(A)** *SALM4* mRNA expression levels were measured in a dose-dependent manner after transfection of HUVECs with three different *SALM4* siRNAs (si*SALM4* #1**−**3) or control siRNA (siCon). **(B)** *SALM4* mRNA expression levels were measured in a time-dependent manner after transfection of HUVECs with two different *SALM4* siRNAs (si*SALM4* #2 and #3) or siCon. **(C)** SALM4 protein expression levels in HUVECs transfected with si*SALM4* #3 were measured by western blot. **(D)** Cells were counted in SALM4-silenced HUVECs under VEGF-A treatment. **(E, F)** Three types of *SALM4* siRNA were evaluated for wound-healing migration (E) and quantification (F). ***P < 0.001 by paired, two-tailed Student *t* test. Error bars represent mean ± SD.

**
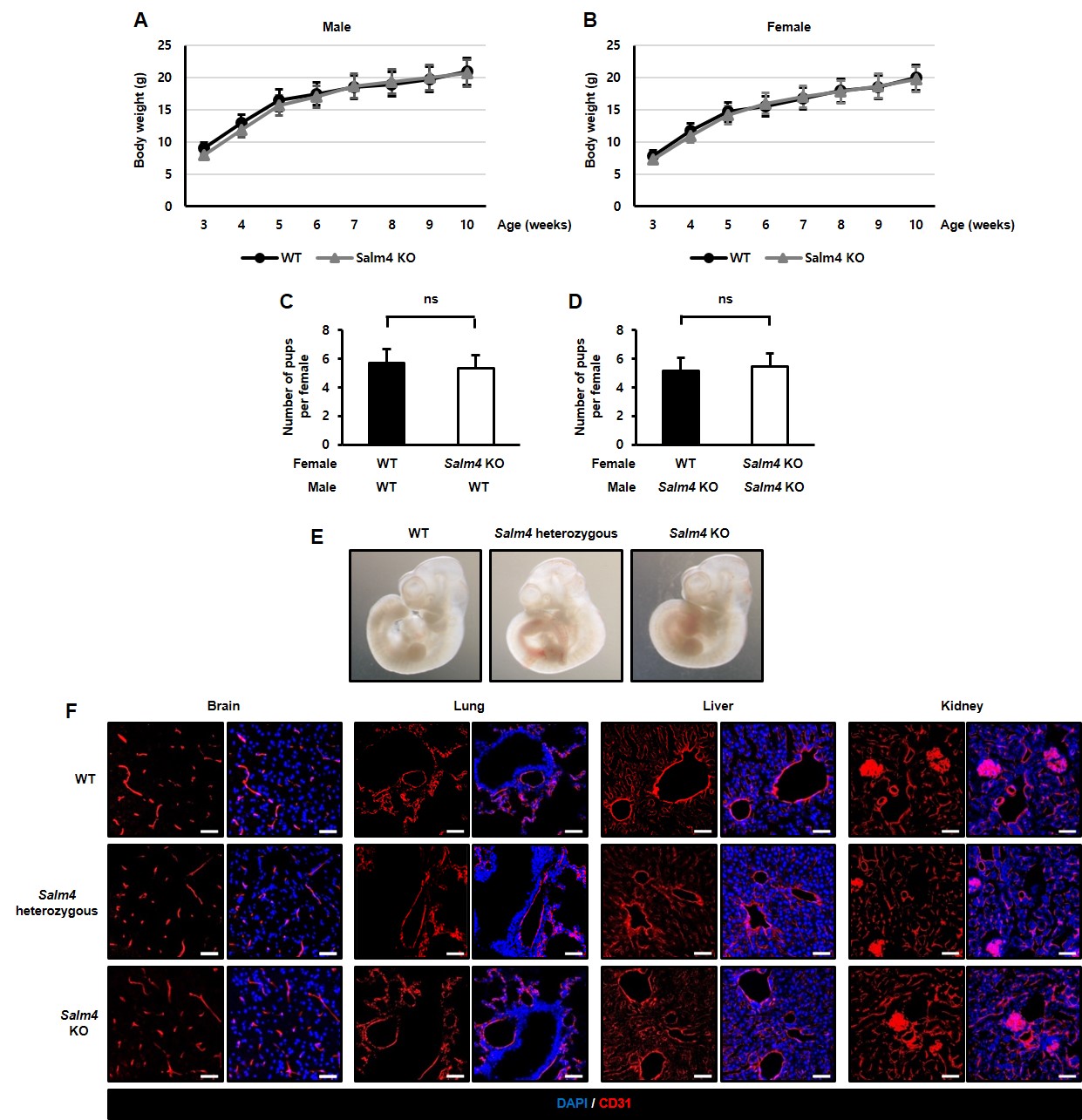
**

**Figure S3. Body weight, fertility, development, and organ vasculature of mice are not affected by *Salm4* knockout.**

**(A, B)** Body weights of WT and *Salm4^−/−^* males (A) or females (B) were recorded from 3-to 10-week-old (n = 7 per group). **(C, D)** WT (C) or *Salm4^−/−^* (D) males (n = 5) aged > 10-week-old were bred to WT or *Salm4^−/−^* females (n = 8) as indicated, and the number of pups was determined over time. **(E)** WT, *Salm4^+/−^*, and *Salm4^−/−^* embryos were photographed on embryonic day 10.5. Each embryo was isolated from embryo conceptus with its yolk sac. n = 5 per group. **(F)** Immunostaining of brain, lung, liver, and kidney sections from 6-week-old WT, *Salm4^+/−^*, and *Salm4^−/−^* mice: nucleus, DAPI; blood vessels, CD31. Scale bars: 50 μm. n = 5 per group. Error bars represent mean ± SD. ns, not significant.

**
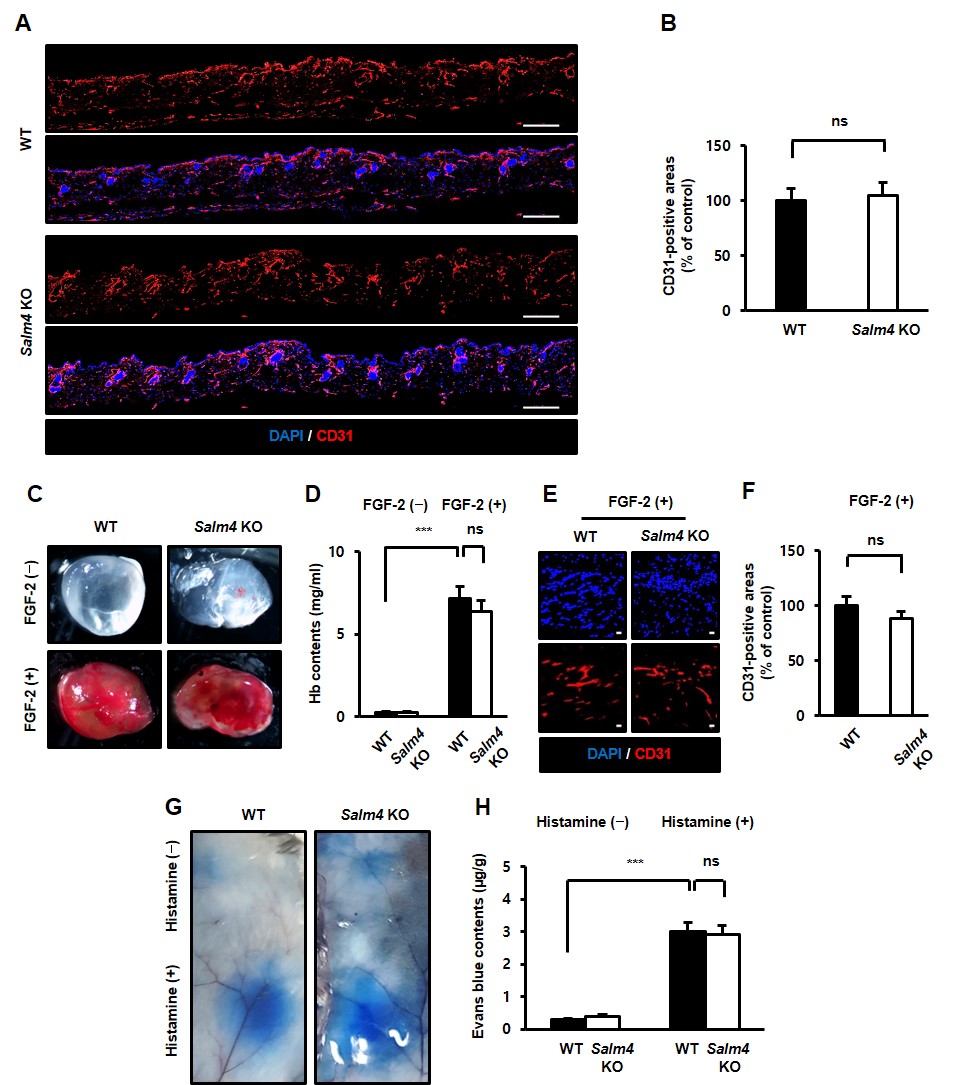
**

**Figure S4. *Salm4^−/−^*** **mice exhibit FGF-2-stimulated angiogenic sprouting and histamine-stimulated vascular permeability.**

**(A, B)** Skin vessel density of WT and *Salm4^−/−^* mice was measured by immunostaining of skin sections: nucleus, DAPI; blood vessels, CD31 (A); quantification of relative CD31-positive areas (B). Scale bars: 200 μm. **(C)** Matrigel mixed with PBS (FGF-2 (**−**) group) or 200 ng/mL FGF-2 (FGF-2 (+) group) were implanted into mice. Images show the Matrigel plugs at day 7 after implantation. **(D)** Quantification of hemoglobin from Matrigel plugs from WT and *Salm4^−/−^* mice. **(E, F)** Immunostaining of Matrigel sections: nucleus, DAPI; blood vessels, CD31 (E); quantification of relative CD31-positive areas (F). Scale bars: 20 μm. **(G)** EB leakage was assessed 30 min after intradermal injection of PBS (histamine (**−**) group) or 200 nmol/mL histamine (histamine (+) group). Six-week-old mice were used (n = 7 per group). **(H)** EB quantification was measured against the standard curve. ***P < 0.001 by paired, two-tailed Student *t* test. Error bars represent mean ± SD. ns, not significant.

**
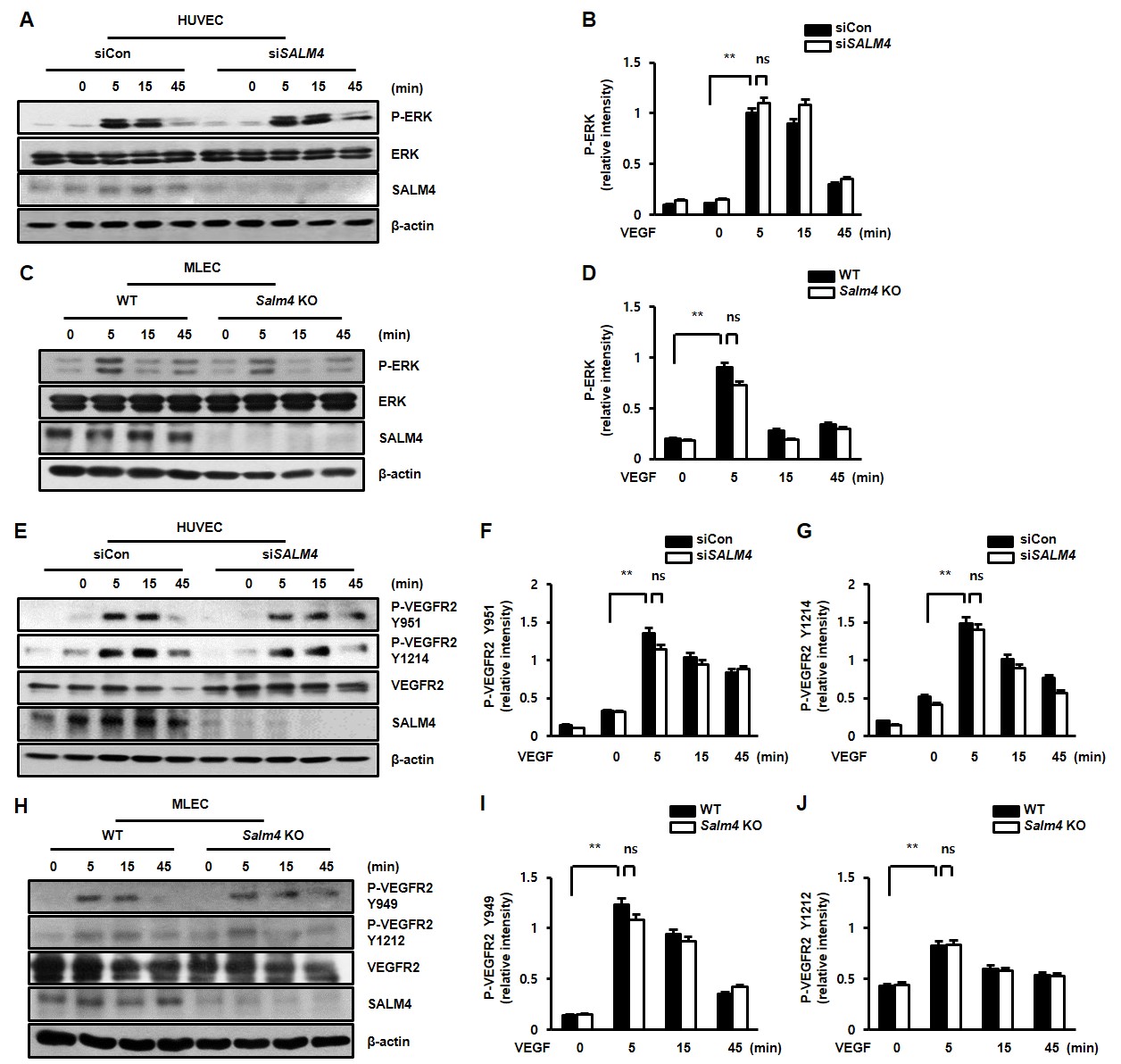
**

**Figure S5. SALM4-silenced ECs do not show altered ERK phosphorylation or VEGF-A-induced VEGFR2 phosphorylation at Y951 (Y949 in mice) and Y1214 (Y1212 in mice).**

**(A)** Effects of SALM4 knockdown on VEGF-A-induced ERK phosphorylation in HUVECs. **(B)** Quantification of blots using Image J software; n = 3 independent experiments. **(C)** Effects of *Salm4* knockout on VEGF-A-induced ERK phosphorylation in MLECs. **(D)** Quantification of blots using Image J software; n = 3 independent experiments. VEGF-A (20 ng/mL) treatment of HUVECs and MLECs. **(E)** Effects of SALM4 knockdown on VEGF-A-induced VEGFR2 phosphorylation in HUVECs. **(F, G)** Quantification of blots using Image J software; n = 3 independent experiments. **(H)** Effects of *Salm4* knockout on VEGF-A-induced VEGFR2 phosphorylation in MLECs. **(I, J)** Quantification of blots using Image J software; n = 3 independent experiments. VEGF-A (20 ng/mL) treatment of HUVECs and MLECs. **P < 0.01 by paired, two-tailed Student *t* test. Error bars represent mean ± SD. ns, not significant.

**
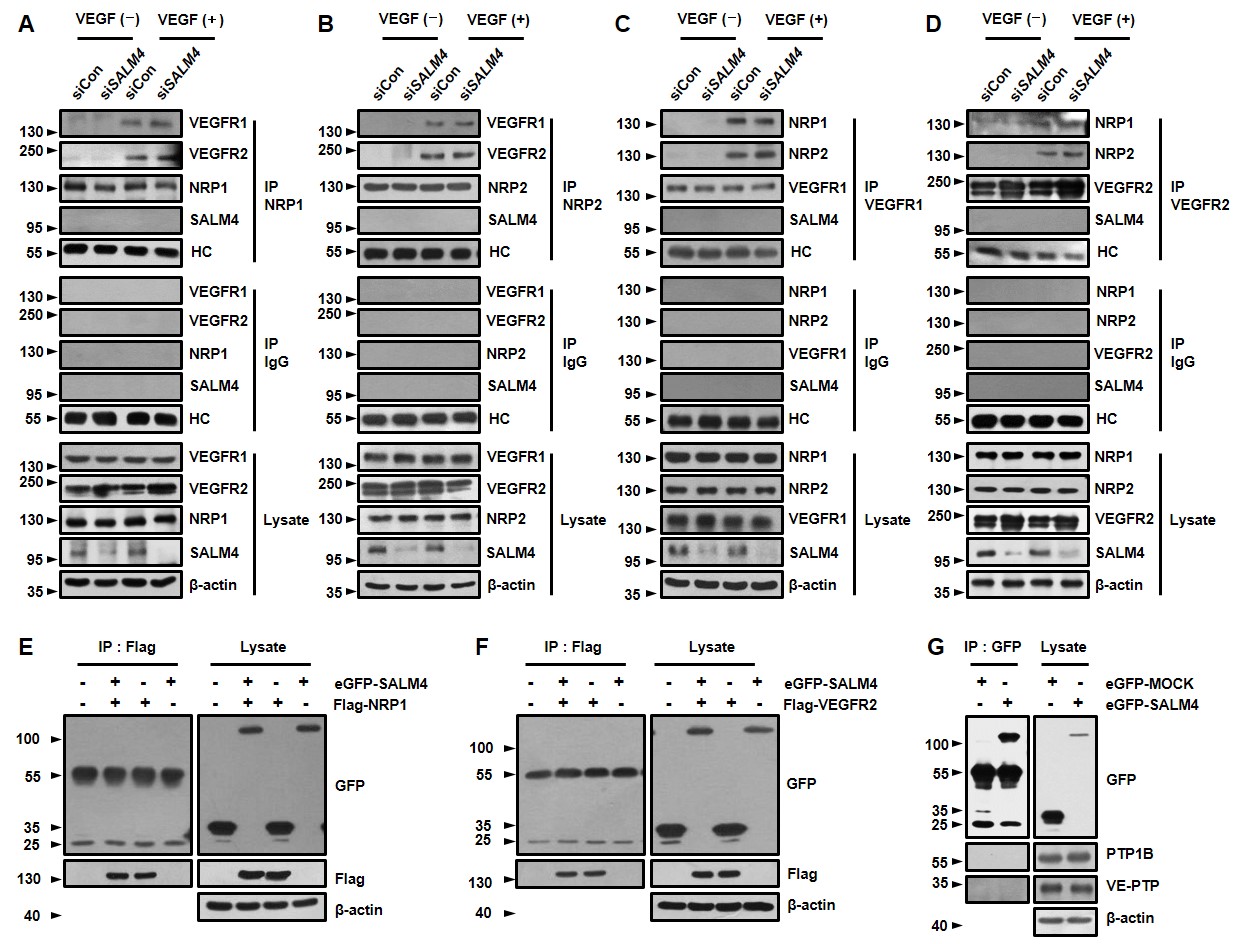
**

**Figure S6. SALM4-silenced HUVECs with VEGF-A stimulation results in VEGFR1/NRP1 or NRP2 and VEGFR2/NRP1 or NRP2 complexes formation.**

**(A, B)** SALM4-deficient HUVECs were stimulated with 30 ng/mL VEGF-A for 5 min, immunoprecipitated with NRP1 (A) or NRP2 (B) antibodies, and blotted for endogenous VEGFR1, VEGFR2, NRP1, NRP2, and SALM4. **(C, D)** SALM4-deficient HUVECs were stimulated with 30 ng/mL VEGF-A for 5 min, immunoprecipitated with VEGFR1 (C) or VEGFR2 (D) antibodies, and blotted for endogenous NRP1, NRP2, VEGFR1, VEGFR2, and SALM4. **(E, F)** Flag-tagged NRP1 (E) or Flag-tagged VEGFR2 (F) were immunoprecipitated with a Flag antibody and blotted for GFP-tagged SALM4 in HEK293T cells. **(G)** SALM4-overexpressed HUVECs were immunoprecipitated with anti-GFP antibody and blotted for endogenous PTP1B and VE-PTP. The same antibodies were used for IP and Western blot. HC, heavy chain; IgG, normal immunoglobulin G; IP, immunoprecipitation.

**
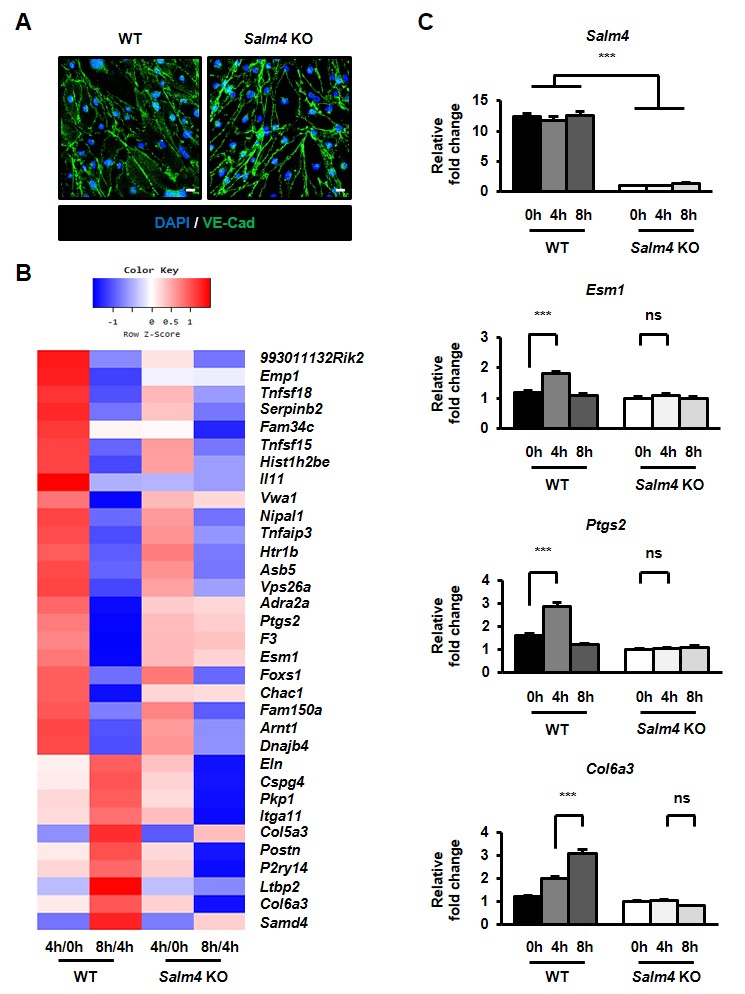
**

**Figure S7. *Salm4^−/−^*** **MLECs show inhibition of angiogenesis-related gene transcription under VEGF-A stimulation.**

**(A)** MLECs were immunostained for VE-cadherin to confirm ECs. Scale bars: 20 μm. **(B)** Heat map of the one-way hierarchical clustering (33 genes) using z-score for fold change. MLECs were isolated in a time-dependent manner and treated with VEGF-A (20 ng/mL). The color gradient indicates fragments per kilobase million (FPKM) +1 and z-score transformations across samples. In the heat map, red represents high expression and blue represents low expression. **(C)** qPCR analysis of mRNA expression levels of known VEGFR2 and PI3K-AKT signaling downstream genes. ***P < 0.001 by paired, two-tailed Student *t* test. Error bars represent mean ± SD. ns, not significant.

**
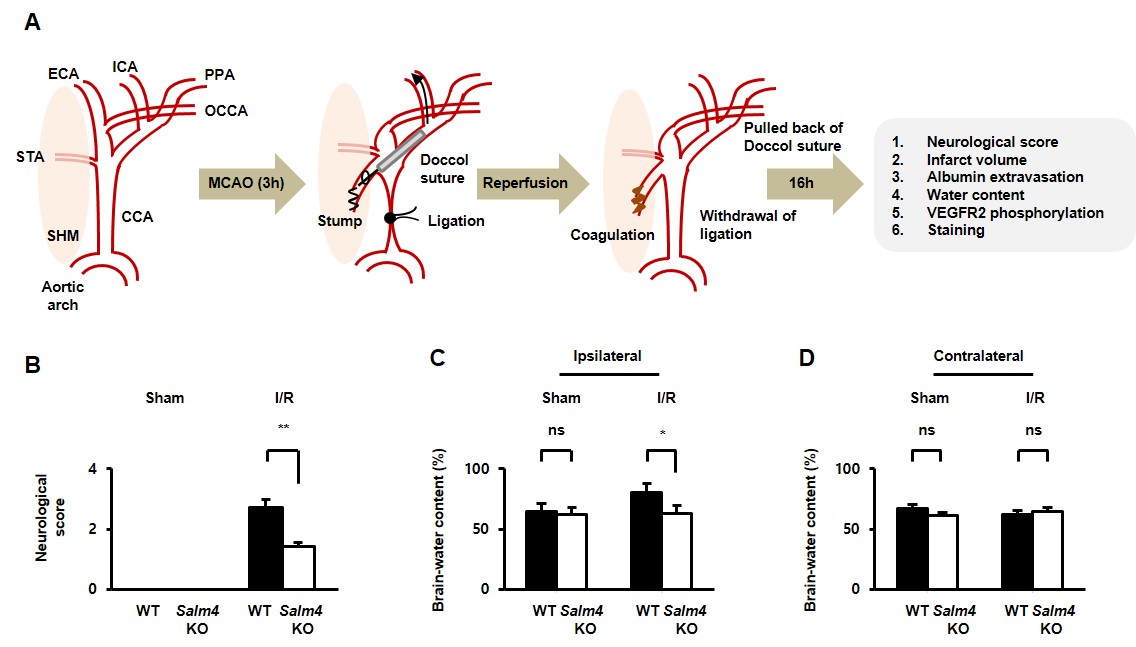
**

**Figure S8. *Salm4^−/−^*** **mice show attenuated neurological scores and edema after I/R.**

**(A)** Schematic drawing of I/R model. A Doccol suture was inserted through the ECA and forwarded into the ICA to occlude the MCA for 3 h. Reperfusion was performed by withdrawing the Doccol suture for 16 h. **(B)** Neurological scores for sham and I/R groups for WT and *Salm4^−/−^* mice. **(C, D)** Brain-water content for sham and I/R groups for WT and *Salm4^−/−^* mice. Quantification of water content in the ipsilateral (C) and contralateral hemispheres (D); n = 7 per group. *P < 0.05, **P < 0.01 by paired, two-tailed Student *t* test. Error bars represent mean ± SD. ns, not significant. CCA: common carotid artery. ECA: external carotid artery. ICA: internal carotid artery. OCCA: occipital artery. PPA: pterygopalatine artery. SHM: sternohyoid muscle. STA: superior thyroid artery.

**
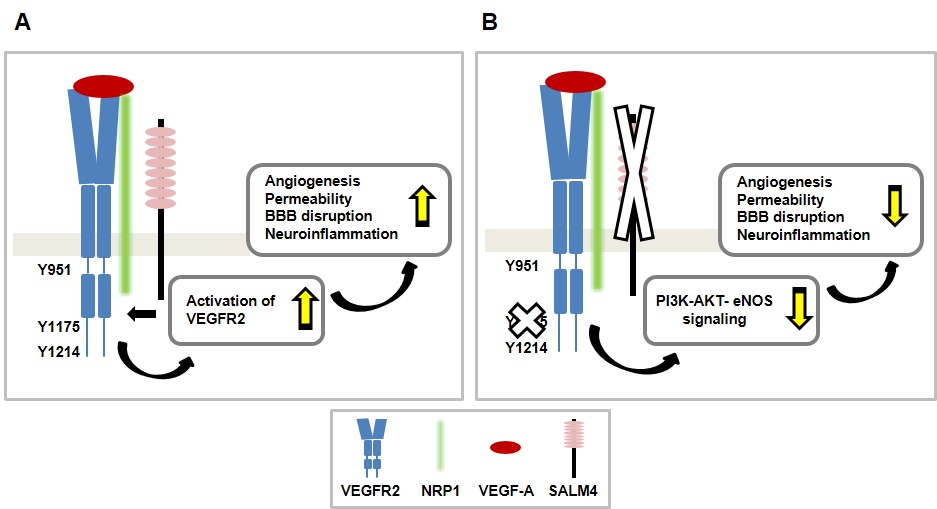
**

**Figure S9. Schematic diagram of SALM4 function in VEGFR2-Y1175 signaling.**

**(A)** SALM4 regulates VEGFR2 phosphorylation and its downstream signaling, modulating angiogenesis, permeability, BBB disruption, and neuroinflammation. **(B)** SALM4 depletion reduces VEGFR2 phosphorylation at Y1175 and its downstream signaling; PI3K-AKT-eNOS. Angiogenesis, permeability, BBB disruption, and neuroinflammation are decreased in SALM4-depleted ECs and *Salm4^−/−^* mice.
